# Do citizens support the transformation of urban transport? Evidence for the acceptability of parking management, car lane conversion and road closures from a German case study

**DOI:** 10.1007/s11116-023-10398-w

**Published:** 2023-05-31

**Authors:** Martin Lanzendorf, Annabell Baumgartner, Nora Klinner

**Affiliations:** Department of Human Geography, Goethe University Frankfurt, Theodor-W.-Adorno-Platz 6, Frankfurt a.M, 60629 Germany

**Keywords:** Urban transformation, Transport policy, Acceptability, Parking management, Cycling network, Road closures

## Abstract

Facing the challenges of motorised traffic, many cities around the globe started implementing measures to transform their urban transport systems. One of the major challenges for the success of adequate policies is not only their effectiveness but also whether they are accepted by city residents. With a quantitative case study in four neighbourhoods in Frankfurt am Main (N = 821), this article investigates the acceptability of three measures: (i) parking management, (ii) the conversion of car lanes into cycle lanes and (iii) the closure of an inner city arterial road to car traffic. The results show a surprisingly high acceptability for all measures if the benefits for local residents are tangible. Thus, successful policy packages may combine push measures with either pull measures, as suggested frequently in the literature, or with improvements for other land uses (e.g. re-using former car-parking spaces for non-transport purposes, such as greenery or seating areas). Furthermore, the perceived effectiveness, daily travel practices and intentions to reduce car use, the built environment and, to a lesser degree, socio-demographics explain differences in acceptability by population group.

## Introduction

Many policymakers and researchers emphasise the need to transform the current car-dominated mobility system to accommodate both the needs of today’s and future generations, to mitigate climate change, land degradation and the exploitation of fossil fuel resources and to improve urban quality of life (UNFCCC 2015; WBGU 2016; Geels et al. 2017; Brand et al. 2021). This transformation has already started in many urban areas worldwide, for example with the growing importance of non-motorised modes, in particular cycling, improved public transport services, the rise of digital and sharing options (e.g. MaaS – Mobility as a Service) and, most importantly, the limitation of and reduction in private car use, e.g. through parking management, road pricing, etc. (Gehl 2010; Aldred et al. 2019; Lanzendorf et al. 2022).

However, daily travel practices as well as planning and policy decisions by local authorities remain dominated by the prevalence of the private car. Despite well-known successes in other ‘best practice’ cities, policymakers often hesitate to implement effective strategies to reduce car dependence, since they are afraid that local residents and other stakeholders will not accept them (Bratzel 1999; Ryghaug and Toftaker 2016; Kirschner and Lanzendorf 2020b).

This gap between the effectiveness and acceptability of transport-related policies is a challenge for policymakers and researchers. For example, Steg (2003) and Harms and Probst (2008) argue that people often readily accept ‘pull’ measures, i.e. measures that improve and increase the attractiveness of alternatives to the private car, but these measures are only to a lesser degree effective for a modal shift away from the private car. Conversely, ‘push’ measures, those that reduce the attractiveness of private car use by increasing the costs and time needed for driving or parking, are far more effective in their impact on modal shift. Unfortunately, individuals are less likely to accept these measures, since they involve higher costs or travel times as well as a perceived restriction of their personal freedom to move (Steg 2003). Thus, many researchers recommend combinations of ‘push’ and ‘pull’ strategies to accomplish a modal shift that is both effective and acceptable (Steg 2003; Gärling and Schuitema 2007; Börjesson et al. 2012).

So far, many case studies have focused on monetary transport measures. Our study, in contrast, examines measures that focus more on the redistribution of public space. It is the objective of this article to analyse the public acceptability of transport-related policy measures in an urban setting. All investigated measures aim to improve the quality of life in urban neighbourhoods, but also contribute to modal shifts by promoting non-motorised, public and shared modes, by reducing the space allocated to the private car and by simultaneously redistributing public space for other transport or non-transport uses. We focus on three urban transport policy measures combining ‘push’ and ‘pull’ elements to reduce the use of private cars and increase the use of alternative modes: (i) the expansion of parking management with increased prices and the redistribution of on-street parking space for other purposes, (ii) the conversion of car lanes into cycle lanes and (iii) the closure of an inner-city, four-lane road section to car traffic and its conversion for non-motorised transport and other non-transport uses.

The selection of these measures in the case study was inspired by ongoing public discussions, media coverage and efforts in the city of Frankfurt am Main for a transformation of the urban transport system. The local government implemented all three measures at least temporarily in some small areas in Frankfurt am Main between 2020 and the end of 2021 when we conducted a quantitative survey to assess the residents’ support for these. However, it was not the aim of this study to evaluate the acceptance of these particular early implementations. Instead, we wanted to assess, more generally, how the local population perceived the potential expansion of these measures either to their own residential neighbourhood (for parking management and lane conversions) or permanently (in the case of the inner city road closure).

For each of the three measures, we will not only assess their acceptability by the population, but also develop a framework for explaining the differences in the levels of acceptability using four factors: (i) the perceived effectiveness of the different measures, (ii) travel practices and intention to reduce car use, (iii) the built environment and (iv) socio-demographics. It should be noted that the three measures took place within the same time period and within the same city, but were not part of a consistent strategy by the local government or other stakeholders (cf. more details in section 3.1). Thus, this article will analyse and compare the factors and their effects for each of the measures separately.

The remainder of this article is structured as follows. In section 2, we outline the related state of the art and, in section 3, the case study and the methodology employed. Next, the results are discussed in sections 4 and 5. In section 4, we analyse the acceptability of different measures and, in section 5, factors explaining the differences in acceptability between various population groups. Section 6 discusses the findings and, ultimately, the paper ends with some conclusions.

## Literature review: factors affecting the acceptability of transport policies

Public acceptability is a key precondition for the successful implementation of transport policies (Steg 2003; Kallbekken et al. 2013) and, consequently, for the transformation of cities. We understand acceptability as an (affirmative) attitude towards a measure and thus – in contrast to ‘acceptance’ – the term does not comprise a behavioural reaction but an evaluation of the expected outcomes of a measure (Schade and Schlag 2000; Schuitema and Steg 2008). Previous findings indicate that the perceived effectiveness, perceived fairness and personal outcome expectations are among the most important factors to explain the acceptability of a measure (Schade and Schlag 2003; Eriksson et al. 2008; Andor et al. 2020).

If a person expects the implementation of a transport policy to be effective at reducing environmental or travel-related problems, he or she is more likely to be in favour of the policy. Nevertheless, this relationship may be causal in both directions (Eliasson and Jonsson 2011). Rienstra et al. (1999), for example, point out that a general rejection of a policy can lead to strategic answers from respondents and, thus, to a lower perceived effectiveness. Similarly, Bolderdijk et al. (2017) argue that an individual’s perception of negative personal consequences may reduce the perceived effectiveness, an effect they label ‘effectiveness skepticism’.

The perceived fairness and personal outcome expectations of a transport policy are related to an individual’s daily travel practices and intention to reduce car use, the built environment and socioeconomic characteristics. For example, regular cyclists or public transport users are more often in favour of a reduction in car infrastructures and regular car drivers are more reluctant towards this (Andor et al. 2020; Kirschner et al. 2020). Similarly, the absence of a private car or a driver’s licence increases the acceptability of congestion charges or taxes (Eliasson and Jonsson 2011; Nilsson et al. 2016). Hence, we assume that car-oriented travel practices, like regular car use as well as permanent car availability, have a negative impact on the acceptability of the transport policies being investigated. Regular use of alternative mobility options, like cycling, walking and public transport, on the other hand, is expected to positively affect acceptability.

Furthermore, daily car travel may be less important to explain the acceptability of measures than the subjective intention to reduce car travel. While a person’s expectation to reduce his/her own car use due to a measure seems to have only a limited effect (Schuitema et al. 2010, b), the willingness to reduce personal car travel in general, independently of a specific local transport policy measure, may increase the acceptability of related measures (Jakobsson et al. 2000; Bamberg and Rölle 2003; Eriksson et al. 2006). Similarly, in a German case study, Kirschner and Lanzendorf (2020b) distinguish between three types of car owners using the stage model of self-regulated behavioural change (Bamberg 2013): (i) those who are very much devoted to and partly dependent on frequent car travel (‘predecision stage’); (ii) those who are considering reducing their car use, but are, as yet, undecided as to how to achieve this goal (‘preaction/action stage’); and (iii) others who have already limited and reduced their car use despite still owning a car (‘postaction stage’). For the last type, the acceptability of suggested parking management measures was significantly higher than for the first one (Kirschner and Lanzendorf 2020b). Thus, we expect people who intend to reduce their personal car use to be more supportive of transport related policy measures aiming to reduce car traffic.

The built environment is both the outcome of and the precondition for the automobile society and structures we find in cities today. Ewing and Cervero (2010) summarise that the built environment can be characterised by so-called ‘5d’ variables: density, diversity, design, destination accessibility and distance to transit. Since the individually expected outcomes of urban transformation processes differ depending on a neighbourhood’s built environment (Westin et al. 2016), we expect the acceptability of transport measures to vary as well. Kirschner and Lanzendorf (2020b), for example, found surprisingly high support for restrictive parking measures in a dense urban environment in Frankfurt am Main. Eliasson and Jonsson (2011) and Winslott-Hiselius et al. (2009) provide evidence, with the example of Stockholm (Sweden), that road pricing is less popular in suburban areas than in more centrally located neighbourhoods. Since the built environment elements in cities are highly complex and it is difficult to discern the effects of single elements, we focus on residential neighbourhoods with different characteristics, like population density, type of housing, distance to the inner city and accessibility of other areas by travel mode. Though we assume that the residential neighbourhood is a decisive factor in a resident’s acceptability of transformation measures, we do not claim that this is merely a causal effect, since residential self-selection effects are at play as well (e.g. Cao et al. 2009).

Finally, the influence of sociodemographic factors on acceptability has already been demonstrated in some cases, but varies between the policies analysed. Most frequently, a significant correlation was found between income and attitudes towards monetary instruments, with lower income having a negative effect on the advocacy of parking or congestion charges (Nilsson et al. 2016; Andor et al. 2020; Kirschner and Lanzendorf 2020b). Jakobsson et al. (2000) and Bamberg and Rölle (2003) also showed that people with a lower income are more likely to perceive the introduction of road pricing as unfair and, thus, less acceptable than people with a higher income, as they feel pressured to reduce their car use due to rising travel costs. Other studies suggest that women are more negative than men towards monetary measures, such as congestion charges (Eliasson and Jonsson 2011) or fuel taxes (Kallbekken et al. 2013; Andor et al. 2020). This might be influenced by differences in the perceived fairness of pricing strategies (Andor et al. 2020), since women have less access to more expensive travel options due to, on average, lower salaries (Kawgan-Kagan 2020). Car-free city centres, on the other hand, seem to be more acceptable to female respondents (Polk 2003; Andor et al. 2020) potentially because women tend to be more environmentally concerned and feel less threatened if a measure questions a “stereotypical view of car use” (Polk 2003). Furthermore, older people approve higher parking fees and road use charges more often than younger people do (Odeck and Kjerkreit 2010; Andor et al. 2020; Kirschner and Lanzendorf 2020b), while the acceptability of improved infrastructure for e-mobility is higher in younger age groups (Andor et al. 2020). Finally, respondents with a higher educational background are more likely to accept transport policies such as congestion charges, parking fees and fuel taxes (Eliasson and Jonsson 2011; Kallbekken et al. 2013; Kirschner and Lanzendorf 2020b). Hence, we assume that income, gender, age and education influence the acceptability of the transport policies being investigated. Nevertheless, the effects of sociodemographic variables on attitudes towards transport policies are comparatively small in some studies (Schade and Schlag 2003; Eliasson and Jonsson 2011; Nilsson et al. 2016).

**Figure 1:**
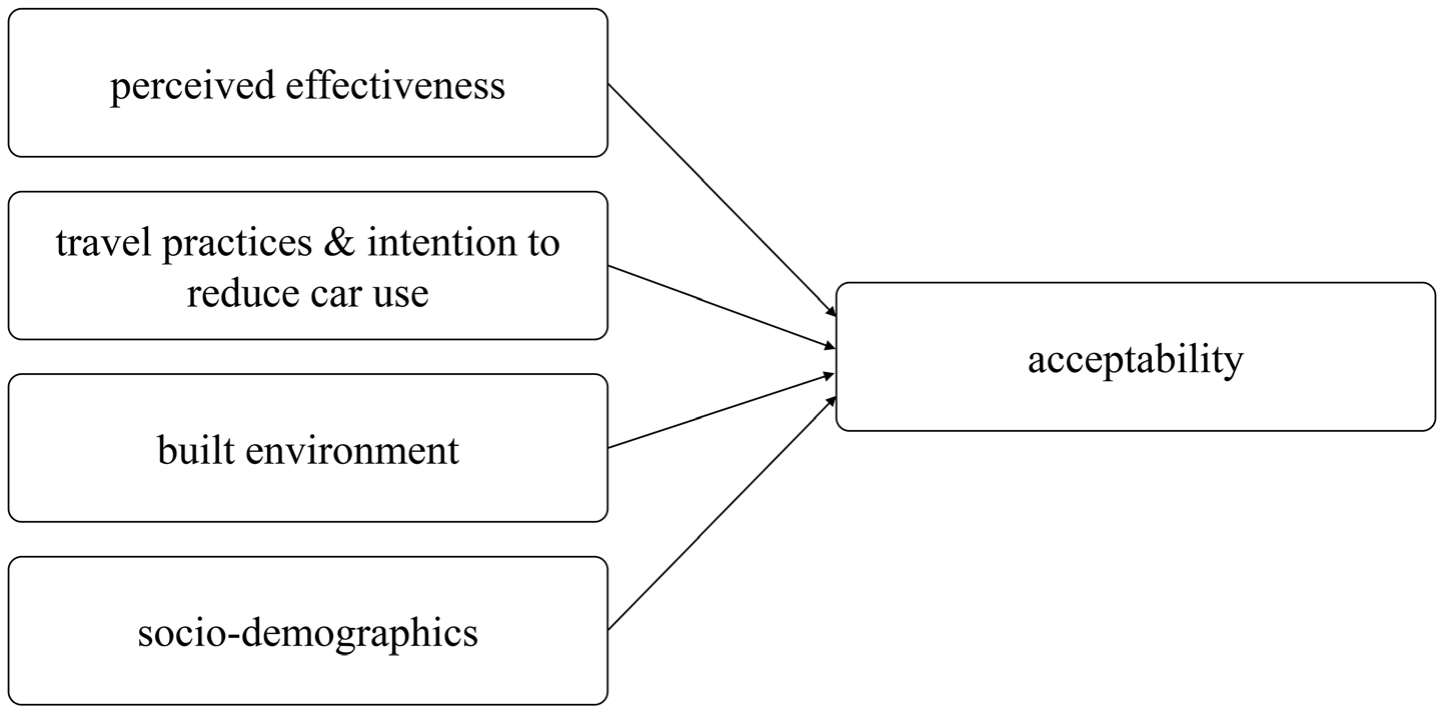
Conceptual model of relevant factors affecting the acceptability of selected urban transformation measures (source: own figure)

From the earlier research, we derived four main groups of factors affecting the acceptability of local transport measures: perceived effectiveness, travel practices and intention to reduce car use, the built environment and socio-demographics (figure 1). Though some strands of the literature suggest mere ‘subjective’, decision-based theoretical frameworks for this endeavour, we opted for a more integrative conceptual framework also taking into account social constructs that are more relevant for daily policy making in cities, like daily travel practices, the built environment and socio-demographics. In our framework, we combined these with the ‘subjective’ factors of perceived effectiveness and the intention to reduce car use. However, it should be noted that we did not include the subjective perceived fairness or the personal outcome expectations in the framework as some other studies did. Though we believe this would enrich future results, we were not able to include all relevant items in our survey.

## Methods

### Case study

Frankfurt am Main, the heart of the German banking and economic system, is located in the western centre of the country with approximately 760,000 inhabitants (as at 31/12/2021). In the last two decades, the city introduced stepwise schemes to strengthen alternatives to the car, mainly cycling and public transport. The modal share of cycling increased from 9% to 2003 to 20% in 2018 (Ahrens et al. 2004; Gerike et al. 2020). Despite policy programmes and ambitions, the changes in transport infrastructures remained relatively limited and small in scale in the city until approximately 2018/19, when at least three (more or less independent) triggers accelerated the actual changes: a Hessian state administrative court decision, a local cycling campaign ‘Radentscheid Frankfurt’ and the objective of the local government to reduce car traffic in the inner city.

First, the Hessian state administrative court (VG Wiesbaden 2018) decided that the emission thresholds for nitrogen oxides had been exceeded for several years and forced the city of Frankfurt to introduce and extend systematic car parking management in the city to meet the objectives of European clean air policies (e.g. European Parliament 2016). Until 2018, restrictions for on-street parking in Frankfurt am Main were rather limited compared to other international examples (Kirschner and Lanzendorf 2020a). Parking fees were limited to the shopping district in the inner city and some other small areas. Even residential parking permits were only issued in inner-city neighbourhoods. With the court decision, restrictive parking regulations became mandatory in Frankfurt am Main. Subsequently, the local government strived to disseminate the new parking regulations starting with neighbourhoods closer to the inner city. In the future, there is an ambition to charge all cars parking on the street in Frankfurt am Main. Non-residents will need a parking ticket; residents still have the option of a cost-reduced parking permit, but this will become more costly than it was in the past. In addition, the new local government also agreed to reduce the total amount of available on-street parking and convert the space for other uses (Koalitionsvertrag 2021). However, this type of new parking management had by the end of 2021 only been implemented in some small areas of the inner city and it remains unclear how long it will take to extend it to the whole city.

Second, the local cycling campaign ‘Radentscheid Frankfurt’, a grassroots movement, successfully initiated a petition for improved cycling infrastructure, safety and related objectives. After its overwhelming support by residents (more than 35,000 signatures within 3 months), almost all the political parties in the city of Frankfurt am Main and the local government agreed to support the cycling movement’s demands. As a result, the local government adopted an ambitious plan ‘Bicycle City Frankfurt am Main’ in 2019 shifting policy objectives further away from the private car and strengthening non-motorised modes. Among the actions being taken, the conversion of car lanes into cycle lanes on different arterial roads was expected to be highly controversial in the local media. However, after the implementation of one of the first lanes in 2020, controversy was limited. Instead, local residents appreciated the improvements to their quality of life (Lanzendorf et al. 2022) and cyclists benefited from the improved safety and quality of infrastructure.

**Map 1:**
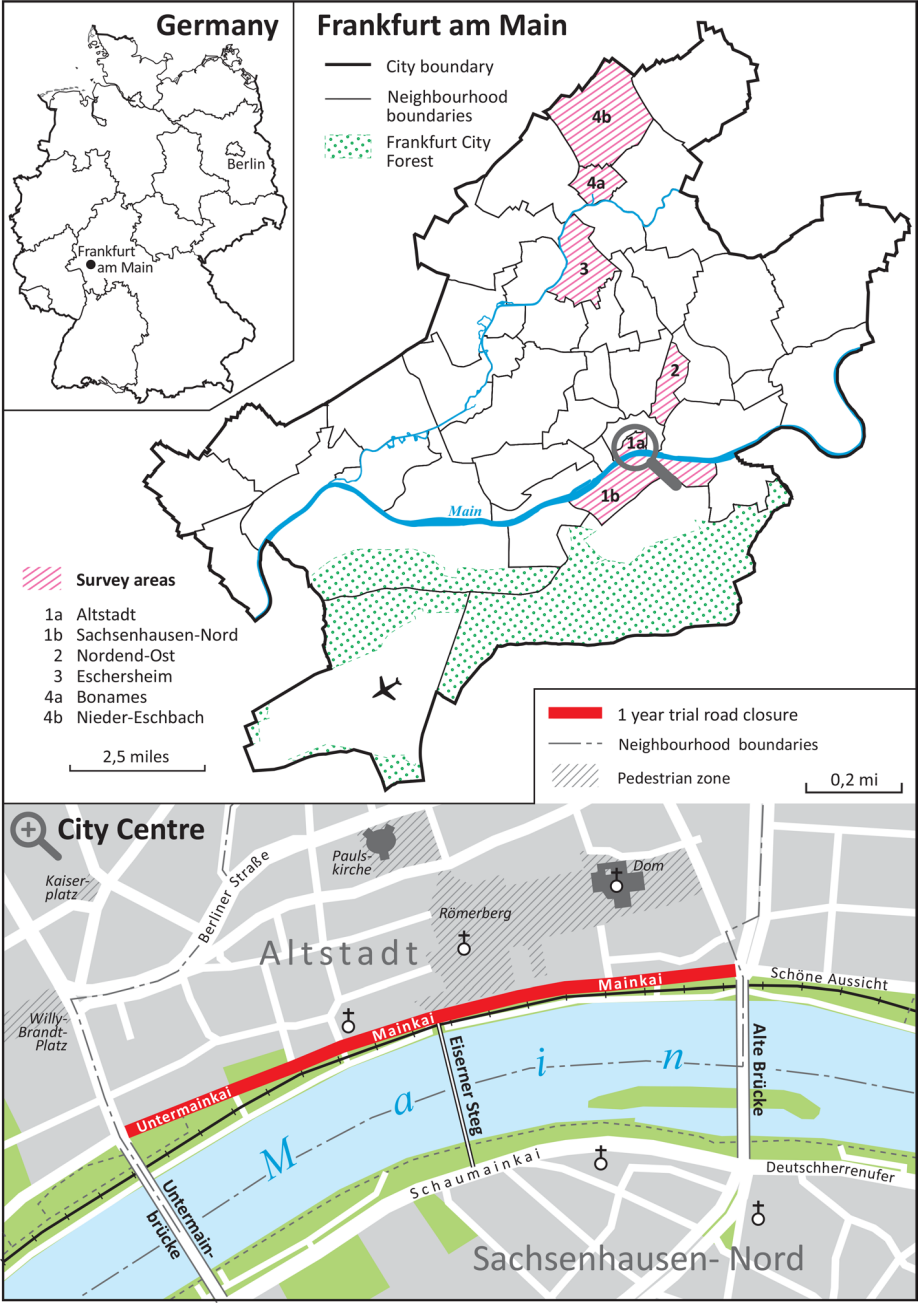
Neighbourhoods surveyed in Frankfurt am Main

Third, for the objective to reduce car traffic and available road space in the inner city of Frankfurt am Main, the local government chose to close one main road in the inner city, the Mainkai, to car traffic. Located on the north bank of the river Main, the Mainkai passes close to the cathedral in the old inner city of Frankfurt am Main (map 1). Local residents in this area have complained about noise, air pollution, safety concerns and a low quality of stay for a long time, but their citizens’ initiatives did not succeed in reducing car traffic despite being supported by various NGOs and political parties. Ultimately, the local government decided on a field trial on the Mainkai for one year between 29 July 2019 and 31 August 2020. The road closure encompassed a road section of 700 m in length with about 20,000 cars per day (Koalitionsvertrag 2016; Pfeiffer-Goldmann 2019).

While the new parking management and the car lane to cycle lane conversions yielded some controversies amongst the public, that became less important after the implementation of the planned projects (for similar observations, see Winslott-Hiselius et al. 2009; Schuitema et al. 2010a; Eliasson and Jonsson 2011), the closure of the Mainkai to car traffic became one of the most controversial issues in local politics in 2020. During the trial in 2019/20, another local residents’ initiative expressed a strong opposition to the Mainkai closure. The residents of the neighbouring district Sachsenhausen-Nord feared that traffic in their neighbourhood might increase, since car drivers needed to find detours. Thus, both initiatives, politicians and other stakeholders pronounced their viewpoints in very controversial ways in public. Ultimately, the field trial ended without an extension before the local elections at the end of 2020 (for some evaluation results, see Pandit et al. 2020). After these elections, despite the new local government’s transport and urban development strategy, the earlier urban transport transformation efforts continued and the future of the Mainkai closure remains unclear at the time of writing this article. Meanwhile, planning initiatives, workshops and temporary closures (weekends, summer vacation) are exploring other types of use.

It has to be noted that, by the time of our survey, the local Frankfurt government did not have a cohesive strategy for combining the three measures (parking management, lane conversions, Mainkai road closure) to achieve an urban transport transformation. Despite many synergies between the measures and an overlapping of many stakeholders involved, each of the measures followed its own rationalities, objectives and time horizons. Thus, with our study we did not aim for an assessment of a (potential but non-existent) cohesive strategy, but limited our survey to the assessment of each measure on its own.

### Survey neighbourhoods

We conducted a quantitative, written survey in four Frankfurt am Main residential neighbourhoods in November and December 2020. Our rationale for the neighbourhood selection was as follows: (i) for all three measures, trials were located in inner city areas (approximately a radius of 3 km from the city centre); (ii) earlier work suggested relatively high acceptability of parking management policies in inner city neighbourhoods (Kirschner and Lanzendorf 2020b) and we wanted to compare the inner city with non-inner city neighbourhoods; (iii) the Mainkai neighbourhood (Altstadt) as well as the close-by neighbourhood of Sachsenhausen-Nord; (iv) the car lane to cycle lane conversion in the Friedberger Landstraße was an important milestone of local transformation policies (Lanzendorf et al. 2022), thus, we wanted to include the close-by neighbourhood of Nordend-Ost; (v) in Eschersheim a local debate emerged in 2020 about parking management in non-inner city neighbourhoods, since residents feared an increase in ‘park & ride’ commuters who benefited from the cost-free parking in their neighbourhood; and (vi) our budget limited the survey to only four neighbourhoods with at least 200 respondents in each.

Eventually, in coordination with local stakeholders and the head of Frankfurt’s transport department, we decided to choose two inner city and two non-inner city neighbourhoods in a sector from the inner city to the North/Northwest of Frankfurt (map 1). In the inner city, we merged the neighbourhoods of Altstadt and Sachsenhausen-Nord to one survey neighbourhood and included Nordend-Ost as the second. Third, we included the non-inner city neighbourhood of Eschersheim and, fourth, merged the neighbourhoods of Nieder-Eschbach and Bonames at the urban fringe to have at least some social stratification in our sample. Bonames is one of the few Frankfurt neighbourhoods with high-rise residential buildings and a low social status, while Nieder-Eschbach is a rather affluent neighbourhood with detached houses.

The selected neighbourhoods represent different built environments, social and transport structures (table 1). The inner city neighbourhoods of Altstadt/Sachsenhausen-Nord and Nordend-Ost are rather densely populated, with a high diversity of functions, excellent public transport supply and good cycling conditions as well as relatively low car ownership rates, while these characteristics are different in the non-inner city survey neighbourhoods. With the exception of Bonames, all of the neighbourhoods surveyed are relatively affluent, though residents with lower incomes or lower social status do live in these neighbourhoods due to the provision of public housing. Eschersheim is similar in its population structure to Nieder-Eschbach, but the inner city is far better accessible by non-car modes: the distance is shorter and, additionally, four subway lines with frequent services pass through Eschersheim, while Nieder-Eschbach is only accessible by one subway line. Moreover, although there is also a mix of detached houses and apartment buildings, the housing stock in Eschersheim is more densely populated.

**Table 1:** Characteristics of the statistical districts surveyed in Frankfurt am Main

	Altstadt	Sachsenhausen-Nord	Nordend-Ost	Eschersheim	Bonames	Nieder-Eschbach	Frankfurt am Main
*extent of residents being affected by Frankfurt policy measures*^*1*^:							
(1) parking management	++	++	++	-	--	--	n.a.
(2) conversion of car lanes to cycle lanes	++	++	++	-	--	--	n.a.
(3) Mainkai closure	++	++	+	--	--	--	n.a.
*built environment*							
distance from city centre	0 km	1 km	3 km	6 km	9 km	11 km	n.a.
population density^2^	84.0	77.5	151.0	47.4	46.6	18.1	30.6
diversity (functional mix)^3^	++	+	+	--	--	-	n.a.
availability of free on-street car-parking^4^	--	--	--	+	++	++	n.a.
short distance to transit^5^	++	++	++	++	++	++	n.a.
*travel practices*							
cars/1000 inhabitants^6^	496	435	356	469	457	733	543
*social*							
needs-based social benefits (%)^7^	12.1%	5.7%	7.3%	8.3%	16.8%	14.8%	12.3%

^1^ ++ measure is/was already implemented in (neighbouring) district, + measure planned in (neighbouring) district, - local debate about measure in district, -- no local debate about measure in district (Stadt Frankfurt am Main 2022)

^2^ population density per hectare (Stadt Frankfurt am Main 2021: 66)

^3^ supply situation in districts measured by the retail and centre concept of Frankfurt am Main: ++ main centre, + local district centre, - basic supply centre, -- no supply centre (Kruse et al. 2018: 101)

^4^ ++ available (almost) everywhere, + mostly available, - partly available, -- hardly available anywhere (mainziel 2022)

^5^ access to subway: ++ subway station in district, + train station in district, - bus station in district, -- no station in district (Stadt Frankfurt am Main 2021: 66; mainziel 2022)

^6^ Stadt Frankfurt am Main 2021: 114

^7^ persons entitled to social benefits for subsistence (basic income support for job seekers, social assistance, asylum seekers’ benefits) (Stadt Frankfurt am Main 2021: 150)

n.a. = not applicable

### Survey and data

We distributed 3,150 questionnaires and selected respondents randomly using a well-established combination of a random-route method and a last-birthday selection promising relatively high response rates (e.g. Kirschner 2019; Blitz 2020). A week after residents received the questionnaires, they got a reminder. Respondents had the option of either sending the survey cost-free back by regular mail or participating using an online link to address a wide target group (Dolnicar et al. 2009: 306). Participants were asked to reply within four weeks. Ultimately, 853 people returned the questionnaire, a response rate of 27% (for details, see Baumgartner et al. 2022). The response rates varied between 26% and 30% in three neighbourhoods, but in Bonames/Nieder-Eschbach it was only 20% (table 2). For the purpose of this analysis, we excluded 32 respondents who stated that they either do not live in the four neighbourhoods surveyed or did not answer this question. Thus, 821 questionnaires remained for the analysis.

Compared with statistical figures from the city of Frankfurt am Main, our sample reflects age and gender structure well despite some variations by neighbourhood. However, income and education qualifications in our sample are relatively high compared to the city of Frankfurt am Main for two reasons: (i) we did not aim for a representative sample of Frankfurt residents but for different degrees of being affected by the urban transformation process that, as yet, mainly focus on inner city areas and where a high share of well-educated people live; and (ii) the underrepresentation of lower income and less educated people in quantitative surveys is a well-known methodological bias (Lepper 2021, see footnote to table 2). Only Bonames/Nieder-Eschbach has, on average, lower incomes and lower education qualifications than the other neighbourhoods and, additionally, the response rate is lower (table 2).

**Table 2:** Socio-demographics and travel characteristics of the sample by neighbourhood

		Altstadt/Sachsen- hausen-Nord		Nordend-Ost		Eschersheim		Bonames/Nieder-Eschbach		Frankfurt am Main	
		sample	total	sample	total	sample	total	sample	total	sample	total
		N = 237	N = 37,079	N = 204	N = 23,135	N = 230	N = 15,323	N = 150	N = 17,912	N = 821	758,847
response rate	%	26.3%		27.2%		30.7%		20.0%		27.1%	
male	%	46.7%	49.0%	48.7%	48.1%	49.3%	48.3%	47.7%	48.8%	47.9%	49.6%
age (years)	mean	49.2	46.5	46.6	46.2	56.9	49.1	55.1	50.5	49.2	47.56
higher education entry qualification	%	83.0%	n.a.	88.8%	n.a.	82.0%	n.a.	64.4%	n.a.	80.8%	58.3% ^2^
net equivalent income^1^ (€)											
< 1000	%	3.6%	n.a.	1.6%	n.a.	2.1%	n.a.	5.0%	n.a.	2.9%	10.1%^5^
1000 - <2000	%	23.2%	n.a.	27.9%	n.a.	21.4%	n.a.	28.1%	n.a.	24.8%	30.0%^5^
2000 - <3000	%	8.2%	n.a.	13.1%	n.a.	8.9%	n.a.	12.4%	n.a.	10.4%	29.9%^5^
3000 - <4000	%	25.8%	n.a.	22.4%	n.a.	30.7%	n.a.	26.4%	n.a.	26.4%	16.0%^5^
4000 - <5000	%	17.0%	n.a.	17.5%	n.a.	19.3%	n.a.	14.9%	n.a.	17.4%	7.1%^5^
> 5000	%	22.2%	n.a.	17.5%	n.a.	17.7%	n.a.	13.2%	n.a.	18.1%	6.9%^5^
*travel practices and car availability* ^*3,4*^											
regular car use	%	47%	n.a.	48%	n.a.	73%	n.a.	80%	n.a.	61%	64%^5^
regular public transport use	%	47%	n.a.	43%	n.a.	46%	n.a.	43%	n.a.	45%	52%^5^
regular bicycle use	%	61%	n.a.	64%	n.a.	51%	n.a.	37%	n.a.	55%	51%^5^
regular walking	%	97%	n.a.	95%	n.a.	83%	n.a.	82%	n.a.	90%	n.a.
car availability	%	64%	n.a.	62%	n.a.	82%	n.a.	82%	n.a.	72%	72%^5^

^1 ^income calculated as OECD-modified equivalence scale, which assigns a value of 1 to the head of household, of 0.5 to each additional adult member and of 0.3 to each child < 14 (OECD n.d.)

^2 ^source: microcensus 2019 for Frankfurt, however the City of Frankfurt reports that in their survey “Living in Frankfurt” 70.3% of respondents had the higher education entry qualification (Lepper 2021: 58)

^3 ^regular mode use was measured on a five dimensional ordinal scale: (almost) daily, 1–3 times per week, 1–3 times per month, less than once a month, (almost) never

^4 ^car availability was measured on a three dimensional ordinal scale: anytime, occasionally, never

^5^ source: Stadt Frankfurt am Main – Bürgeramt, Statistik und Wahlen, 2022 (regular mode use was measured on a five dimensional ordinal scale: daily, several times per week, several times per month, less frequently, never)

Source:own survey (2020); Frankfurt data provided by the statistics office City of Frankfurt/Main (31.12.2020, only citizens aged ≥ 18); n.a. = not available.

We assessed the perceived effectiveness of the three measures discussed using 19 items: five for parking, seven for the conversion of car lanes into cycle lanes and seven for the road closure of the Mainkai (table 3). It is remarkable that the perceived effectiveness of the road closure to car traffic and the lane conversion are higher than for the parking management. For the latter, the perceived effectiveness is close to the neutral score for all items. Obviously, the lane conversions and the road closure immediately change the street layout and, thus, the respondents expect the effects of more cycling and fewer cars to be quality of stay and noise reduction. However, the effects of different parking management are much less obvious, since they are rather long-term and will remain limited if the street layout does not change and the motorised traffic does not decrease simultaneously.

We conducted a principal component analysis (PCA) with all 19 items. With the Meyer-Olkin criterion, we derived three factors explaining 70.1% of the variance of these items: (i) perceived effectiveness of parking policies, (ii) perceived effectiveness of a road closure to car traffic and (iii) perceived effectiveness of a conversion of car lanes into cycle lanes (table 3). It should be noted that we conducted one PCA resulting in three factors that map the initial three groups of indicators.

**Table 3:** Principal Component Analysis (PCA) of the items measuring perceived effectiveness of the proposed measures

	mean	sd	Component 1 ‘perceived effectiveness of parking policies’	Component 2 ‘perceived effectiveness of a road closure to car traffic’	Component 3 ‘perceived effectiveness of a conversion of car lanes into cycle lanes’
*parking policies*^*1*^: I think that if such policies were implemented in my living environment, …					
… the noise level would decrease.	0.12	1.41	0.879	0.191	0.137
… the air quality would improve.	0.09	1.40	0.862	0.197	0.152
… the quality of stay would increase.	0.29	1.35	0.849	0.230	0.184
… the number of car trips would decrease in the long term.	0.13	1.38	0.813	0.196	0.217
… the safety of people walking or cycling would increase.	0.35	1.39	0.801	0.229	0.230
*road closure to car traffic*: I think a street closure to cars at Mainkai and a permanent redesign of the area would …					
… increase the quality of stay along the river Main.	1.17	1.15	0.166	0.870	0.249
… make walking more pleasant.	1.21	1.09	0.151	0.855	0.242
… decrease the noise level.	1.06	1.12	0.252	0.788	0.252
… improve air quality.	0.90	1.22	0.290	0.759	0.270
… promote cycling.	0.91	1.16	0.307	0.650	0.424
… reduce the number of car trips in the long term.	0.15	1.31	0.426	0.530	0.334
… make car driving less convenient in Frankfurt.	0.84	1.09	− 0.071	− 0.265	− 0.118
*conversion of car lanes into cycle lanes*: Along the route, I think a conversion would …					
… make cycling more pleasant.	1.33	0.96	0.079	0.314	0.834
… increase the safety of cyclists.	1.28	1.01	0.093	0.326	0.817
… reduce the number of conflicts between road users.	0.20	1.38	0.260	0.188	0.677
… decrease the noise level.	0.53	1.31	0.544	0.249	0.601
… improve air quality.	0.59	1.32	0.502	0.275	0.594
In Frankfurt, I think a conversion would …					
… promote cycling.	1.13	1.10	0.207	0.333	0.743
… reduce the number of car trips in the long term.	0.38	1.36	0.422	0.348	0.576
Cronbach’s alpha			0.941	0.833	0.910

PCA with varimax rotation; only factors with eigenvalues > 1 were considered; loadings < 0.4 are shown in grey; N = 821; Kaiser-Meyer-Olkin = 0.930; Bartlett’s test of Sphericity = 11750.55, df = 171, p = 0.000; Total variance explained: 70.1%; all items were measured on a five-point Likert scale with 2 (strongly agree), 1 (slightly agree), 0 (neither agree nor disagree), -1 (slightly disagree), -2 (strongly disagree)

^1^ This item was preceded by a statement: ‘In many cities, parking areas for cars are being restructured. This often means that some of the parking spaces are reserved for residents with parking permits. Fees are charged for the remaining parking spaces to pay for the expansion of cycle lanes and public transportation. In addition, some of the parking spaces will be converted into greenery, wider pavements or bicycle parking spaces, for example.’

Source: own survey (2020).

Regarding daily travel practices, respondents were asked about the frequency of their car, public transport, bicycle and walking mode use respectively. Individuals are considered a regular user of a particular mode if they use it at least once a week (table 4). In addition, respondents are classified as car users whether they have a car at their disposal at all times or not. The respondents’ stage in the SSBC for reducing car use (Bamberg 2013) was determined using a set of four indicators on a Likert scale (see table 4). The aim was to assign each respondent with these indicators to one of the four stages of predecision, preaction, action and postaction. If respondents agreed with more than one statement indicating different stages, we assigned the highest one to them in terms of readiness to reduce car use (e.g. a respondent agreeing on predecision and preaction was assigned to the preaction stage). In addition, the 63 respondents who did not assign themselves to any stage but do not own a car, never have a car available and (almost) never use a car were assigned to the postaction stage. The remaining 4% of cases were not assigned to any of these stages but assigned a missing value for the stage model. Since the group of those in the preaction or action stage was relatively small (90 cases), these two groups were combined into one transition phase.

**Table 4:** Definition and descriptive statistics of travel practices, intention to reduce car use and residential neighbourhood

	description	% of respondents
*travel practices* ^*1,2*^		
regular car use	1 = car driver or passenger at least weekly	60.5%
regular public transport use	1 = pt use at least weekly, 0 = less frequently	54.6%
regular bicycle use	1 = bicycle use at least weekly, 0 = less frequently	44.7%
regular walking	1 = walking at least weekly, 0 = less frequently	89.9%
car availability	1 = a car is available at any time, 0 = no	71.8%
*intention to reduce car use* ^*3*^		
predecision stage^4^	1 = yes, 0 = no	25.7%
preaction or action stage^5^	1 = yes, 0 = no	11.4%
postaction stage^6^	1 = yes, 0 = no	62.9%
*residential neighbourhood*		
Altstadt/Sachsenhausen-Nord	1 = yes, 0 = no	28.9%
Nordend-Ost	1 = yes, 0 = no	24.8%
Eschersheim	1 = yes, 0 = no	28.0%
Bonames/Nieder-Eschbach	1 = yes, 0 = no	18.3%

^1^ regular mode use was measured on a five dimensional ordinal scale: (almost) daily, 1–3 times per week, 1–3 times per month, less than once a month, (almost) never

^2 ^car availability was measured on a three dimensional ordinal scale: anytime, occasionally, never

^3^ the stage model of self-regulated behavioural change (SSBC) was measured on a five-point Likert scale: strongly agree, slightly agree, neither agree nor disagree, slightly disagree, strongly disagree and the answers were grouped into 3 binary variables of stage allocation

^4^ ‘I am satisfied with my car use and see no need to change it.’

^5^ ‘At the moment I use the car a lot. However, I am considering driving less. I am not yet sure whether and how I can achieve this goal./I already know exactly how to achieve this goal, I just need to put my plan into action.’

^6 ^‘I have made a conscious decision to use other means of transport instead of the car as often as possible. In the future, too, I will maintain my low car use or reduce it even further.’

Source: own survey 2020 (N = 821).

## Results 1: the acceptability of policy measures by the urban population

To measure the acceptability of policy measures (table 5), we employed items on a five-point Likert scale regarding parking management already used and tested in a previous study (Kirschner and Lanzendorf 2020b). To assess the acceptability of car lane to cycle lane conversion as well as the Mainkai closure, we developed items that were similar to the parking items. The items were discussed with experts from the research group as well as with practitioners from the Frankfurt transport department. Furthermore, all items were tested in a pretest. From all these discussions and testing, we did not have any indication of reliability or validity issues.

Considering the controversies in the local media regarding some of the measures being investigated, as well as the reluctance of local politicians to implement such measures and their concerns about facing opposition, the acceptability of all three groups of measures is surprisingly high (table 5). However, the level of agreement differs between the measures studied. Respondents strongly support the idea of converting the dominant car infrastructure into alternative land uses, either for non-motorised travel modes or for non-transport uses. More than two thirds of respondents agree with the conversion of car lanes into cycle lanes, 59% with the re-use of on-street parking for cycle lanes and 68% and 64% respectively with an improvement in cycling and pedestrian conditions on the Mainkai.

This positive attitude towards non-motorised modes is also reflected, among other things, in the acceptability of the Mainkai road closure to car traffic. 71% of the respondents support the idea of converting the inner-city, four-lane road in the future. 60% believe this measure to be necessary, but there is a strong polarisation of opinions regarding its closure to cars: 45% approve this but almost the same amount of respondents disapprove. We understand this polarisation as a result of the ongoing and at times polemic public discussions between the supporters and opponents of the measure during the experimental closure. Though some of these different opinions may be attributed to the residential location and how it was affected by the Mainkai closure (section 5), we believe that the public dispute was driven by more general questions relating to the future role of the private car in society and in Frankfurt’s inner city as well. Despite their low agreement on the closure to cars in the future, respondents would be more likely to agree if, additionally, some road space were converted to non-transport uses, like green spaces (73%), seating (69%), outdoor gastronomy (61%), areas for music or theatre events (59%), playgrounds (52%) or sports (48%) and, to a lesser degree, shared urban gardening (44%) or retail stalls (30%).

In the case of parking management, the package with combined measures finds a relatively high level of support among citizens (56%), despite the high conflict potential of parking in urban policymaking. However, it should be noted that only one fifth of respondents agrees to an introduction of parking fees alone in their own residential neighbourhood. This is not surprising, noting that in Frankfurt am Main, as in most German cities, parking has been free for a long time with the exception of the inner city (section 3.1). Although this policy is slowly changing, it might still take some time before citizens get used to this.

The support for converting on-street parking spaces to other uses depends strongly on the alternatives. The highest agreements for conversions of parking spaces are to cycle lanes (59%) and urban green spaces (52%), to a lesser degree to bicycle parking (43%), wider pavements (41%), seating areas (37%), outdoor areas for gastronomy (36%), carsharing parking (34%), playgrounds (31%) and delivery services (23%).

**Table 5:** Acceptability of different transport policy measures

	total (N)	strongly agree (%)	slightly agree (%)	neither agree nor disagree (%)	slightly disagree (%)	strongly disagree (%)	mean	sd
*parking policies*								
I think the measures described are desirable for my neighbourhood.^1^	807	34.0	22.1	16.4	10.9	16.7	0.46	1.47
I think fees should be charged for all parking spaces in my neighbourhood.	803	12.7	6.6	12.6	15.4	52.7	-0.89	1.43
I would approve if parking spaces in my neighbourhood were transformed into …								
… cycle lanes.	798	39.1	20.1	11.3	9.0	20.6	0.48	1.56
… greenery.	799	34.2	18.3	10.9	10.6	26.0	0.24	1.63
… bicycle parking spaces.	790	22.4	21.0	18.2	11.3	27.1	0.00	1.52
… wider pavements.	795	22.4	18.4	18.9	15.1	25.3	-0.03	1.50
… seating areas.	793	17.0	19.8	20.3	13.9	29.0	-0.18	1.47
… outdoor areas for gastronomy.	786	14.2	21.6	19.7	15.4	29.0	-0.23	1.43
… parking spaces for car sharing vehicles.	781	12.2	21.5	23.8	15.5	27.0	-0.24	1.37
… playgrounds.	788	15.5	15.9	19.2	17.5	32.0	-0.35	1.45
… areas for parcel and delivery services.	789	7.9	15.0	27.5	20.9	28.8	-0.48	1.26
*conversion of car lanes into cycle lanes*								
I would like to see car lanes converted into cycle lanes in Frankfurt.	813	47.4	19.8	10.7	6.9	15.3	0.77	1.48
*road closure to car traffic*								
In future, the Mainkai should again be completely closed to car traffic.	792	33.0	11.7	14.4	10.6	30.3	0.06	1.66
I would like to see the Mainkai redesigned in the future.	740	47.8	22.8	14.7	5.0	9.6	0.94	1.30
The Mainkai should offer more space for cyclists in the future.	772	42.4	25.8	14.5	5.3	12.0	0.81	1.35
The Mainkai should offer more space for pedestrians in the future.	783	43.8	20.1	16.2	8.0	11.9	0.76	1.39
In my view, it is necessary to redesign the Mainkai in the future.	774	37.9	22.5	19.3	8.3	12.1	0.66	1.37
I would then be more in agreement with a Mainkai closure to cars if it was also designed and used in the following way:								
greenery	794	50.6	22.3	11.1	4.5	11.5	0.96	1.35
seating areas	790	37.3	32.0	13.8	5.6	11.3	0.79	1.31
outdoor areas for gastronomy	790	30.4	30.1	16.7	9.2	13.5	0.55	1.36
areas for events, e.g. open air stage for music/theatre	785	29.9	29.0	18.0	8.5	14.5	0.51	1.38
playgrounds	777	27.5	24.6	22.5	8.6	16.7	0.38	1.40
sports equipment/sports fields	780	22.7	25.0	23.5	11.8	17.1	0.24	1.38
commonly used garden areas	767	25.8	18.5	20.2	12.1	23.3	0.11	1.50
outside areas for retailers	773	11.9	17.7	26.1	19.4	24.8	-0.28	1.33

All items were measured on a five-point Likert scale with 2 (strongly agree), 1 (slightly agree), 0 (neither agree nor disagree), -1 (slightly disagree), -2 (strongly disagree); ^1^This item was preceded by a statement: ‘In many cities, parking areas for cars are being restructured. This often means that some of the parking spaces are reserved for residents with parking permits. Fees are charged for the remaining parking spaces to pay for the expansion of cycle lanes and public transportation. In addition, some of the parking spaces will be converted into greenery, wider pavements or bicycle parking spaces, for example.’

Source: own survey (2020).

## Results 2: factors explaining variances in acceptability by the population

In order to investigate which factors influence the acceptability of different measures, we calculated regression models for each of the following three measures: (i) the package of parking management measures with increased parking fees and the redistribution of on-street parking spaces for other purposes, (ii) the conversion of car lanes into cycle lanes and (iii) the acceptability of the closure of the inner-city, four-lane Mainkai road to car traffic (table 6).

By testing the assumptions of linear regression models, we found that multicollinearity was met but not the normality of residuals. Since the models’ sample size is relatively large, the small deviation of the residuals from a normal distribution does not necessarily lead to biased results, but should be acknowledged (Schmidt and Finan 2018). To assess the impact of some observed heteroscedasticity, we carried out tests using robust models that showed no differences in the results. Since the sociodemographic variables ‘higher education entry qualification’ and ‘income’ are not significant in all models, we tested other variable specifications (e.g. ‘university degree’, ‘age square’ and ‘income square’ to assess non-linear effects), but our model results remained robust and other specifications of the socioeconomic variables did not deliver any additional explanatory power.

All three regression models show significant results with relatively high explanatory powers (adjusted R² between 0.40 and 0.61). The constant is significant in all models, indicating the relatively high acceptability of all measures, in particular for the conversion of car lanes into cycle lanes.

**Table 6:** Linear regression models for acceptability of three transport policy measures

	parking policies^1^				lane conversion^2^			road closure to car traffic^3^		
	B	β		Sig.	B	β	Sig.	B	β	Sig.
*perceived effectiveness*										
perceived effectiveness parking policies	0.675	0.464		***						
perceived effectiveness lane conversion					0.766	0.521	***			
perceived effectiveness road closure to car traffic								0.752	0.462	***
*travel practices*										
regular car use	-0.059	-0.019			-0.087	-0.029		-0.442	-0.131	***
regular public transport use	0.084	0.028			-0.036	-0.012		0.038	0.011	
regular bicycle use	0.431	0.145		***	0.470	0.157	***	0.397	0.119	***
regular walking	0.162	0.033			0.064	0.013		0.038	0.007	
car availability	-0.222	-0.068		**	-0.361	-0.110	***	-0.076	-0.021	
*intention to reduce car use * *[ref: preaction and action stage]*										
predecision stage	-0.456	-0.134		***	-0.763	-0.223	***	-0.321	-0.084	
postaction stage	0.196	0.064			0.163	0.053		0.216	0.063	
*neighbourhood [ref: Eschersheim]*										
Altstadt/Sachsenhausen-Nord	0.149	0.046			-0.281	-0.087	***	-0.178	-0.050	
Nordend-Ost	0.172	0.051			-0.105	-0.031		0.082	0.022	
Bonames/Nieder-Eschbach	-0.369	-0.098		***	-0.143	-0.038		-0.021	-0.005	
*socio-demographics*										
male	-0.021	-0.007			0.078	0.026		0.025	0.007	
age	-0.008	-0.088		***	-0.003	-0.031		-0.007	-0.074	**
higher education entry qualification	-0.077	-0.021			0.008	0.002		-0.046	-0.011	
income	-8.394E-06	-0.009			-2.266E-05	-0.023		-2.534E-05	-0.024	
constant	0.716			**	1.192		***	0.593		*
R²		0.477				0.612			0.413	
Adjusted R²		0.467				0.605			0.402	
N		821				821			821	
F-statistics		***				***			***	

Regression models with robust standard errors, using HC3 method; missing values are replaced by mean; B: regression coefficient (estimate of the change in the dependent variable that can be attributed to a change of one unit in the independent variable); Beta (β): standardised regression coefficient; significance: *p < 0.10 **p < 0.05 ***p < 0.01

Tests suggest the existence of heteroscedasticity but results from regression with robust standard errors do not differ from those obtained using simple OLS

^1 ^‘I think the measures described are desirable for my neighbourhood.’ (see footnote 1, tables 3 and 5); ^2 ^‘I would like to see car lanes converted into cycle lanes in Frankfurt.’; ^3 ^‘In future, the Mainkai should again be completely closed to car traffic.’

Source: own survey (2020).

In each of the models, the perceived effectiveness of the specific measure is the strongest predictor of variances in acceptability. In the model for parking measures, furthermore, regular bicycle users support the measures more than others and residents with permanent car availability less so. Those not intending to reduce their car use and thus being in the predecision stage also are less inclined to support the potential parking measures. Also, the built environment has a significant effect. Residents living further away from the city centre (Bonames/Nieder-Eschbach) agree with the measures to a lesser degree than those of the more centrally located residential neighbourhoods (Altstadt/Sachsenhausen-Nord, Nordend-Ost and Eschersheim). Finally, most sociodemographic factors are not significant. However, older people are less likely to support the parking measures than younger people.

In the model for the conversion of car lanes into cycle lanes, the effects of regular bicycle use and car availability are similar to the parking measures model. Thus, regular cyclists support the idea of lane conversions to a higher degree than others and residents with permanent car availability to a lesser degree. Furthermore, being in the predecision stage of the SSBC model reduces the acceptability of the lane conversion. Surprisingly, for the built environment, the acceptability pattern does not follow the model for parking measures. For lane conversions, the inner city residents (Altstadt/Sachsenhausen-Nord) agree with the measure to a lesser degree than residents in the other neighbourhoods (Nordend-Ost, Eschersheim, Bonames/Nieder-Eschbach). Socio-demographics are not relevant for this model.

Finally, the regression model for the Mainkai road closure to cars reveals an effect of daily travel practices. Regular car use, unsurprisingly, reduces the acceptability of a road closure to car traffic. Additionally, as in the other two models, regular cyclists are more likely to support this measure. In contrast, the residential neighbourhoods show no effect in the model. Compared to the other neighbourhoods, residents in Altstadt/Sachsenhausen-Nord do not show a higher or lower support for the road closure. This, at first glance, surprising result can only be understood by a more detailed look at the local residents. By dividing the residents in the neighbourhood into those living in the immediate vicinity of the closure on the north bank of the river Main (Altstadt) and those who live on the south side of the river Main (Sachsenhausen-Nord), we find that 64% of the residents in the Altstadt but only 32% in Sachsenhausen-Nord approve the measure. Of the sociodemographic factors, only age is significant. Similar to the parking management model, older people support the road closure less than younger people.

## Discussion

Our results indicate a surprisingly high acceptability of the proposed measures by the local residents. However, the level of agreement differs between the measures studied. More than two thirds of the respondents support the car lane to cycle lane conversions, 56% the parking management package and 45% the Mainkai closure. Furthermore, and similar to previous studies (Steg 2003; Gärling and Schuitema 2007; Börjesson et al. 2012), combinations of ‘push’ and ‘pull’ measures are more popular than ‘push’ measures alone as the support for the package of parking measures (56%) in comparison with parking fees alone (19%) shows (section 4).

In each model, the perceived effectiveness of the specific measure assessed (e.g. effectiveness of parking measures in the parking model) is the strongest predictor of acceptability which is similar to findings reported in earlier studies (Schade and Schlag 2003; Eriksson et al. 2008; Andor et al. 2020). However, it should be noted that this is not necessarily a causal relationship but could also be the result of a reverse causality, the effectiveness skepticism effect, meaning that respondents who oppose a measures tend to perceive it as non-effective (Bolderdijk et al. 2017; see also Rienstra et al. 1999).

Similar to previous findings (Andor et al. 2020; Kirschner 2021), daily travel practices have an influence on the acceptability of transport policies as well. Unsurprisingly, the acceptability of parking measures is particularly high among regular cyclists, as the survey data indicates that they are more aware of the danger posed by parked cars to pedestrians and cyclists^1^. In the case of a road closure to car traffic, this measure is supported by regular cyclists as well, while regular car users are more likely to oppose it.

Furthermore, in line with our preliminary assumption that people with a strong car orientation oppose the suggested changes, our results show that being in the predecision stage of the SSBC model reduces the support for parking management and lane conversion measures. As regular car use does not affect the acceptability of these measures, the individual intention to reduce car use in the future contributes additional explanatory power to the regression models. Similar to the findings of another study in Frankfurt am Main (Kirschner and Lanzendorf 2020b), we conclude that subsequent acceptability research should consider an individual’s intention to reduce car use in the future. However, the SSBC indicators are not significant for the acceptability of the Mainkai street closure to car traffic. Despite not being able to explain this unexpected observation, the heated public discussion around this topic in Frankfurt possibly affects an individual’s reasoning and rationality so that the regular use of a car or a bicycle for daily travel better explains the acceptability in the model than the SSBC indicators.

Whether a redistribution of public spaces is considered necessary and desirable depends, among other things, on the availability and quality of these alternative land uses in the residential neighbourhood. Regarding the parking policies in our survey, residents in dense urban areas approve these measures more frequently than those in more suburban areas, as suggested by the literature (Winslott-Hiselius et al. 2009; Eliasson and Jonsson 2011). However, this explanation is not valid for the other two measures. Despite the lane conversions receiving the highest support in all neighbourhoods on average, the respondents in the core of the city (Altstadt/Sachsenhausen-Nord) agree less frequently to this measure than those in the other neighbourhoods. While this might be caused by the ongoing debates regarding the Mainkai in this neighbourhood and the polarisation of opinions between cyclists and car drivers, another reason might be how the neighbourhood is affected by lane conversions at various places since the local administration focused its first cycling infrastructure developments in this area. Furthermore, the support of the Mainkai closure does not differ between the residential neighbourhoods, indicating that this measure was perceived differently compared to the other two measures, since we did not ask for the acceptability of the road closure in an individual’s own residential neighbourhood but in the city centre.

However, the residents within the Altstadt/Sachsenhausen-Nord neighbourhood had conflicting interests due to diverging outcome expectations of the measure (Westin et al. 2016). As already mentioned (section 3.1), the road closure was controversially being discussed in the media at the time of the survey. While the local government portrayed the closure as a benefit for the residents of Altstadt in terms of, for example, an improved quality of stay, the opponents from the residents’ initiative in Sachsenhausen-Nord claimed negative consequences for their neighbourhood (e.g. increased motorised traffic, increased air and noise pollution). Therefore, the higher approval in Altstadt could be attributed to the residents’ better outcome expectations compared to those from Sachsenhausen-Nord (Schuitema and Steg 2008).

Of the sociodemographic variables, only age becomes relevant in the models. With increased age, opposition to parking management and road closures increases. This is striking, since previous studies that focused on charges for parking and road use suggest that older people are more inclined to these types of measures (Odeck and Kjerkreit 2010; Andor et al. 2020; Kirschner and Lanzendorf 2020b). However, most measures analysed in the present study are non-monetary and focus on the redistribution of public spaces. Hence, they imply changes to the built environment and not only in the user costs of the existing infrastructure. Similarly, Andor et al. (2020) maintain that older people are less in favour of an improved e-mobility infrastructure than younger people and, thus, with the redesign of the built environment. Various authors argue that socialisation processes and generational effects affect daily travel practices differently by age group (Döring et al. 2014; Selzer 2021). Older residents in particular may find it difficult to change their daily car practices, explore new transport options or new layouts of streets and places. Furthermore, with age some physical limitations may occur or be expected in the future so that some daily practices, such as grocery shopping, visiting friends and relatives or recreational activities, may be difficult to imagine without a car after years or decades of car practices (Aguiler and Cacciari 2020). So, the acceptability of measures that aim to redistribute car spaces becomes limited. By contrast, younger residents may be much more open-minded to changes in the urban environment by these measures, partly because of different socialisation processes with a decreasing importance of the car (Chatterjee et al. 2018). However, it should be noted that age was not significant for the conversion of car lanes into cycle lanes. Safe cycling infrastructure as well as a cycling culture may increase cycling, thus, in all age and vulnerable groups (e.g. older people, children, inexperienced cyclists) (Paradowska 2018; Hudde 2022).

Some limitations of our study should be noted. First, the measures examined were discussed differently by local media before and during the data collection, which may have affected the respondents’ perceptions. For example, the Mainkai street closure was discussed controversially by the local media contributing to a strong polarisation of opinions amongst the public. In contrast, the changes in parking management have, so far, received only limited attention from the population in most neighbourhoods. Second, most of the neighbourhoods in our survey have a highly educated and wealthy population. Thus, further and more detailed analyses of less affluent neighbourhoods might enrich our understanding of the residents’ support for the ongoing policies. Third, in our theoretical framework, we included the built environment, travel practices and socio-demographics, but not the personal affectedness nor the knowledge about each measure, two factors frequently mentioned as important for explaining differences in acceptability. Fourth, since the survey took place in winter 2020, the first winter of the Covid-19 pandemic, studies carried out at a different time might lead to different results. For example, the travel practices, attitudes or acceptability of measures may differ from other periods before the pandemic, but may still remain valid for the ‘new normality’ after it.

## Conclusions

Facing the consequences of the climate crisis as well as the negative health and environmental impacts of motorised traffic, many cities around the globe started implementing measures to transform their urban transport systems. One of the major challenges for the successful introduction of adequate policies is not only their effectiveness but also their acceptability by city residents. This article therefore investigated the acceptability of three measures: (i) parking management, (ii) the conversion of car lanes into cycle lanes and (iii) the closure of an inner city arterial road to car traffic.

To assess the local residents’ acceptability of measures for an urban transformation, we developed an integrated theoretical framework. This combines psychological (perceived effectiveness and intention to reduce car use), sociological (age, gender, education, income), spatial (built environment) and transport related (car availability, mode use) factors to improve our understanding of acceptability. Further, the model does not focus on the acceptability of national, regional or city-wide policy measures but on the neighbourhood level.

The results show that all factors of the model contribute to the explanation of acceptability, but perceived effectiveness is the strongest factor in all models. However, this may be due to a reversed causality, meaning that a low perceived effectiveness is not the reason but the consequence of a low acceptability of the measure in question (Bolderdijk et al. 2017). Furthermore, residents’ daily mode use, the intention to reduce car use and the built environment may increase or decrease the acceptability of measures. Since the objectives of most urban transformation policies include the reduction of car use and car infrastructures as well as the improvement of non-motorised modes, public transport systems and the quality of stay in public spaces, we may expect that each step in the direction of the objectives may increase the acceptability of related measures further. For example, an improvement in cycling conditions should increase the number of cyclists and, thus, the acceptability of further car lane to cycle lane conversions. This might become similar to the increased acceptance of congestion charges after implementation compared to beforehand (e.g. Schuitema et al. 2010a; Nilsson et al. 2016). However, this was not analysed in this study but is an interesting challenge for future research.

Regarding policy implications, our results show that not only may ‘pull’ measures strengthen the acceptability of ‘push’ measures but also the re-use of land for non-transport purposes (e.g. improved liveability, redistribution of roads or parking space for other uses). For example, the ‘push’ measures of parking management can be combined with the conversion of parking spaces to other land uses (e.g. greenery, seating areas) or for non-motorised transport (e.g. a cycle lane). Thus, urban transport transformation is not a task for transport planners alone but also for integrative urban and transport planning.

Urban transport transformations are always complex tasks which are difficult to implement. In the case of Frankfurt am Main, the context has been very supportive for urban transformation policies in the last five years. The international movements for mitigating climate change (e.g. Fridays-For-Future, Scientists-For-Future) as well as the local ‘Radentscheid Frankfurt’ campaign were strongly supported by the public. Additionally, the administrative law court decisions regarding the need to reduce car emissions (section 3.1) and the continued success of the Green party in the local elections provided the basis for a fundamental change in local transport policy with the ‘Bicycle City Frankfurt am Main’ decision as a milestone in 2019.

But even if a majority of residents supports specific measures for an urban transport transformation, its implementation may still become difficult if a local group of people vehemently opposes a measure (e.g. local business, residents from adjacent neighbourhoods) and succeeds in forming an alliance with other urban initiatives and stakeholders (e.g. political parties, lobby groups, the media). The example of the Frankfurt Mainkai closure shows that a strong polarisation of residents’ opinions in two adjacent neighbourhoods caused a standstill in political decision making and consensus finding. Possibly the support of local residents for this measure might have increased considerably with better involvement of the public in the planning process (Odeck and Bråthen 1997, 2002), a communication campaign, a convincing plan for the redesign of the road section with alternative transport infrastructures and land uses as well as the opportunity to experience this during a trial period (Schuitema et al. 2010a).

## Data Availability

The datasets generated and analysed during the current study are not publicly available due to data protection requirements. However, they can be made available for further research on reasonable request.

## Footnotes

1 We asked about the perception of dangers posed by parked cars and tested in a t-test whether the perception of dangers by regular cyclists differs from the perception by others. The difference is significant (p < 0.05).
